# ATAD2 promotes glycolysis and tumor progression in clear cell renal cell carcinoma by regulating the transcriptional activity of c-Myc

**DOI:** 10.1007/s12672-023-00696-1

**Published:** 2023-05-26

**Authors:** Zonglong Wu, Liyuan Ge, Yimeng Song, Shaohui Deng, Peichen Duan, Tan Du, Yaqian Wu, Zhanyi Zhang, Xiaofei Hou, Lulin Ma, Shudong Zhang

**Affiliations:** Department of Urology, Peking University Third Hospital, Beijing, 100191 P.R. China

**Keywords:** Clear cell renal cell carcinoma, ATAD2, Glycolysis, c-Myc

## Abstract

Clear cell renal cell carcinoma (ccRCC) is a common malignant tumor of the urogenital tract. Given that ccRCC is often resistant to radiotherapy and traditional chemotherapy, the clinical treatment of patients with ccRCC remains a challenge. The present study found that ATAD2 was significantly upregulated in ccRCC tissues. In vitro and in vivo experiments showed that the inhibition of ATAD2 expression mitigated the aggressive phenotype of ccRCC. ATAD2 was also associated with glycolysis in ccRCC. Interestingly, we found that ATAD2 could physically interact with c-Myc and promote the expression of its downstream target gene, thereby enhancing the Warburg effect of ccRCC. Overall, our study emphasizes the role of ATAD2 in ccRCC. The targeted expression or functional regulation of ATAD2 could be a promising method to reduce the proliferation and progression of ccRCC.

**Supplementary Information:**

The online version contains supplementary material available at 10.1007/s12672-023-00696-1.

## Introduction

Renal cell carcinoma (RCC) is a common malignant tumor of the urinary system, with a global incidence increasing by 2% yearly [1]. Clear cell renal cell carcinoma (ccRCC) is the most common subtype of RCC, which accounts for 75% of all RCC cases [2]. It is well established that early diagnosis and surgical treatment of ccRCC can effectively improve patients’ prognosis. However, a recurrence rate of 20–40% has been reported after nephrectomy [3]. Moreover, ccRCC is prone to metastasis, and approximately one-third of patients present with distant metastasis at diagnosis [4]. Limited treatment options are currently available for advanced ccRCC because of its high resistance rates to traditional chemotherapy and radiotherapy. In ccRCC, cell energy metabolism tends to be aerobic glycolysis rather than oxidative phosphorylation; this phenomenon is termed the Warburg effect [5]. The Warburg effect maintains the energy demand of tumor cells and promotes the growth and metastasis of tumors. Importantly, the serum levels of lactate, the end product of glycolysis, are significantly increased in ccRCC patients, and the expression of glycolysis-related enzymes is also upregulated in ccRCC tumor tissues [6].

As a member of the ATPase family, the ATPase family AAA domain-containing protein 2 (ATAD2) is recently reported to be closely associated with tumor progression. ATAD2 contains two conserved domains, namely the bromine domain and the ATPase domain which are responsible for histone binding and regulation of ATPase activity [7]. ATAD2 expression is upregulated in gastric, ovarian, and breast cancers [8–10]. An increasing body of evidence suggests that ATAD2 promotes cell proliferation and metastasis in tumor occurrence and development [11, 12]. ATAD2 overexpression is usually associated with poor clinical outcomes [13]. However, the expression and relevance of ATAD2 in ccRCC remain largely unclear, thereby warranting further research.

In the present study, we found that the expression of ATAD2 in ccRCC was remarkably upregulated, which induced the malignant phenotype of ccRCC. Mechanistically, ATAD2 mainly promotes the downstream transcription activity of c-Myc by interacting with c-Myc, thereby promoting the glycolysis of ccRCC. Accordingly, the regulation of ATAD2 expression may prevent ccRCC progression and enable its therapeutic treatment.

## Materials and methods

### Identification of differentially expressed genes in ccRCC

To reveal the transcriptomic signature of ccRCC, the microarray datasets GSE66270 and GSE68417 from the Gene Expression Omnibus (GEO) database were used [14]. The GSE66270 dataset contains 14 normal and 14 tumor samples. The GSE68417 dataset contains 14 normal and 29 tumor samples. By using the GEO2R tool analysis, genes showing fold change (FC) > 2 and P < 0.05 in their expression in ccRCC as compared to that in normal renal tissues were identified and defined as differentially expressed genes (DEGs) in ccRCC. The overlapping components were analyzed by Funrich software. The ClusterProfiler software package was used for pathway analysis with the Kyoto Encyclopedia of Genes and Genomes (KEGG) database [15]. Next, The Cancer Genome Atlas (TCGA) data portal was used to obtain gene expression profiles of 530 ccRCC patients, and these profiles were analyzed by the limma package [16].

### Cell culture

ccRCC cell lines (A498, caki-1, 769P, 786-O, and OS-RC2) and the immortalized human kidney cell line HK-2 were purchased from American Type Culture Collection. The cells were cultured in RPMI 1640 medium (Biological Industries) containing 10% fetal bovine serum (FBS). For inhibitor treatment, the cells were stimulated with the ATAD2-specific inhibitor BAY-850 (1 µM, MCE) for 24 h. For c-Myc inhibition experiments, the c-Myc inhibitor 10,058–F4 (100  mmol/L, Selleck) was used to treat ccRCC cells for 24 h.

### Synthesis of small interfering RNA, plasmid construction, and cell transfection

The cells were transfected with specific small interfering RNAs (siRNAs) targeting ATAD2 (si-ATAD2). si-ATAD2 was purchased from Biosynthesis (Beijing, China). The sequences of siRNAs were as follows:

si-ATAD2–1 sense: 5′-GAAUAUUGAUAGUAGGAGATT-3′, antisense: 5′-UCUCCUACUAUCAAUAUUCTT-3′;

si-ATAD2–2 sense: 5′-CUAUACCACUAGUGAGAAATT-3′, antisense: 5′-UUUCUCACUAGUGGUAUAGTT-3′;

si-ATAD2–3 sense: 5′-CUGAUAAAGAGGCUCGAAATT-3′, antisense: 5′-UUUCGAGCCUCUUUAUCAGTT-3′;

si-N. C sense: 5′-UUCUCCGAACGUGUCACGUTT-3′, antisense: 5′-ACGUGACACGUUCGGAGAATT-3′.

The *ATAD2* gene was cloned into the pcDNA3.1 plasmid. According to the manufacturer’s protocol, lipofectamine 2000 (Invitrogen) was used to transfect the plasmid or siRNA. In the in vivo experiment, the recombinant lentiviral plasmid and the packaging plasmid were co-transfected into 293T cells to generate lentiviral particles. After A498 cells were infected with lentivirus, 4 µg/mL puromycin was used for 48 h for selection. The cells were maintained in a medium containing 2 µg/mL puromycin to obtain a stable cell line.

### Cell proliferation analysis

Cell counting kit 8 (CCK-8) and 5-ethynyl-2′-deoxyuridine (EdU) assays were used to evaluate the changes in cell proliferation. A total of 2 × 10^3^ ccRCC cells were inoculated in 96-well plates, and CCK-8 (Beyotime) assay was performed after 1, 2, 3, 4, and 5 days. The EdU kit (Ribobio) was used for EdU measurement in accordance with the manufacturer’s instructions.

### Western blotting assay and antibodies

Proteins were separated using 8–10% sodium dodecyl sulfate-polyacrylamide gel electrophoresis (SDS-PAGE). The separated proteins were transferred to polyvinylidene difluoride (PVDF) membranes (Millipore, Billerica, MA, USA). After blocking with a 5% blocking solution, the PVDF membranes were incubated with primary antibodies overnight at 4 °C. Following incubation with the appropriate secondary antibodies, the Luminata Crescendo Western HRP substrate (Millipore) was used for processing, exposure, and digital imaging.

Supplemental Table S1 provides the list of antibodies used in the experiment.

### Immunohistochemistry and immunofluorescence analysis

Antigens were retrieved from deparaffinized tissue sections, which were then blocked with 5% bovine serum albumin. Tissue sections were incubated with primary antibodies overnight at 4 °C, followed by incubation with peroxidase-conjugated secondary antibodies to detect antigen-antibody complexes. Subsequently, a color reaction was performed using a 3,3ʹ-diaminobenzidine (DAB) substrate kit (ZsBio). ccRCC cells were plated on coverslips and fixed in 4% paraformaldehyde (PFA) for immunofluorescence staining. Next, the cells were treated with 0.25% Triton X-100 for 15 min. After blocking with 5% donkey serum, the coverslips were incubated overnight with primary antibodies at 4 °C. Fluorescent secondary antibodies were used to detect the primary antibodies.

### Immunoprecipitation

IP lysis buffer (20 mM Tris-HCl, 150 mM NaCl, and 1% Triton X-100, pH 7.5) was used for cells lysed. The protein lysis buffer was incubated with antibodies (anti-ATAD2, anti-c-Myc, or IgG as control antibodies) overnight on a shaker at 4 °C, followed by incubation with protein A + G agarose beads (Santa Cruz) for 4 h. Immune complexes were eluted from the agarose beads and subsequently analyzed by SDS-PAGE followed by immunoblotting assay. Light chain-specific secondary antibodies (1:2000, 58,802 S; CST) were used to prevent the IgG heavy chain from obscuring the signal of the target protein. The Flag-ATAD2 and HA-c-Myc plasmids were co-transfected into HEK293T cells by Lipofectamine 2000 (Invitrogen). After 48 h, the cells were lysed with IP lysis buffer. Flag fusion proteins were immunoprecipitated by anti-Flag magnetic beads (Beyotime) and eluted with 3X Flag peptide (Beyotime) and analyzed by SDS-PAGE followed by immunoblotting.

### In vivo animal studies

This study was approved by the Ethics Committee of Peking University Third Hospital (A2023031). A subcutaneous xenograft tumor model was used to evaluate the effects of ATAD2 on tumor growth in vivo. A total of 5 × 10^6^ vector control cells and cells stably expressing ATAD2 were injected subcutaneously into the armpits of nude mice. After 1 week, the subcutaneous tumor volume was calculated with a digital caliper every 7 days by using the following formula: V = width^2^$\times$ length/2. Mice were monitored, at minimum, once every 3 days, and the tumors were not allowed to exceed 1.5 cm in diameter or 1500 mm^3^ in volume. After 4 weeks, the mice were euthanized. The tumor tissues were then weighed and paraffin-embedded for hematoxylin and eosin (H & E) staining and immunofluorescence analysis.

### Silver staining

The Flag-ATAD2 plasmid was transfected into HEK293T cells. The IP lysis buffer was used to prepare the whole cell lysate. Subsequently, the whole cell lysate was immunoprecipitated overnight with Flag magnetic beads or IgG control beads (Beyotime) at 4 °C. The precipitate was then washed with cold IP cleaning buffer for 5 times. In accordance with the manufacturer’s instructions, the rapid silver staining kit (Beyotime) was used to visualize the isolated ATAD2 binding proteins.

### Detection of glucose, adenosine triphosphate, and lactic acid levels

Glucose, lactic acid, and intracellular adenosine triphosphate (ATP) levels were identified with the corresponding detection kits (Nanjing Jiancheng Corporation) in accordance with the manufacturer’s instructions.

### c-Myc green fluorescent protein reporter assays

To monitor c-Myc activity, a c-Myc green fluorescent protein (GFP) reporter plasmid was obtained from Yeasen (11744ES03). The sequence of the c-MYC response element was as follows: GGCCTAACTGGCCGGTACCGCTAGCCTCGATCACGTGCACGTGCACGTGCACGTGGCGCGTAGATCTGCAGAAGCTTAGACACTAGAGGGTATATAATGG.

The c-Myc GFP reporter plasmid, and the ATAD2 plasmid were co-transfected into HEK293T cells for 48 h. The c-Myc pathway activation was evaluated by detecting the GFP with a fluorescence microscope.

### Statistical analysis

All experiments were conducted at least three times. The data are expressed as mean ± standard deviation (SD). Data analysis was conducted using GraphPad Prism 8.0. Statistical analysis was performed using unpaired two-tailed t-test, two-way ANOVA, and one-way ANOVA followed by Tukey’s multiple comparison test. A P-value of < 0.05 was considered statistically significant.

## Results

### Identification of DEGs in ccRCC

**Fig. 1:**
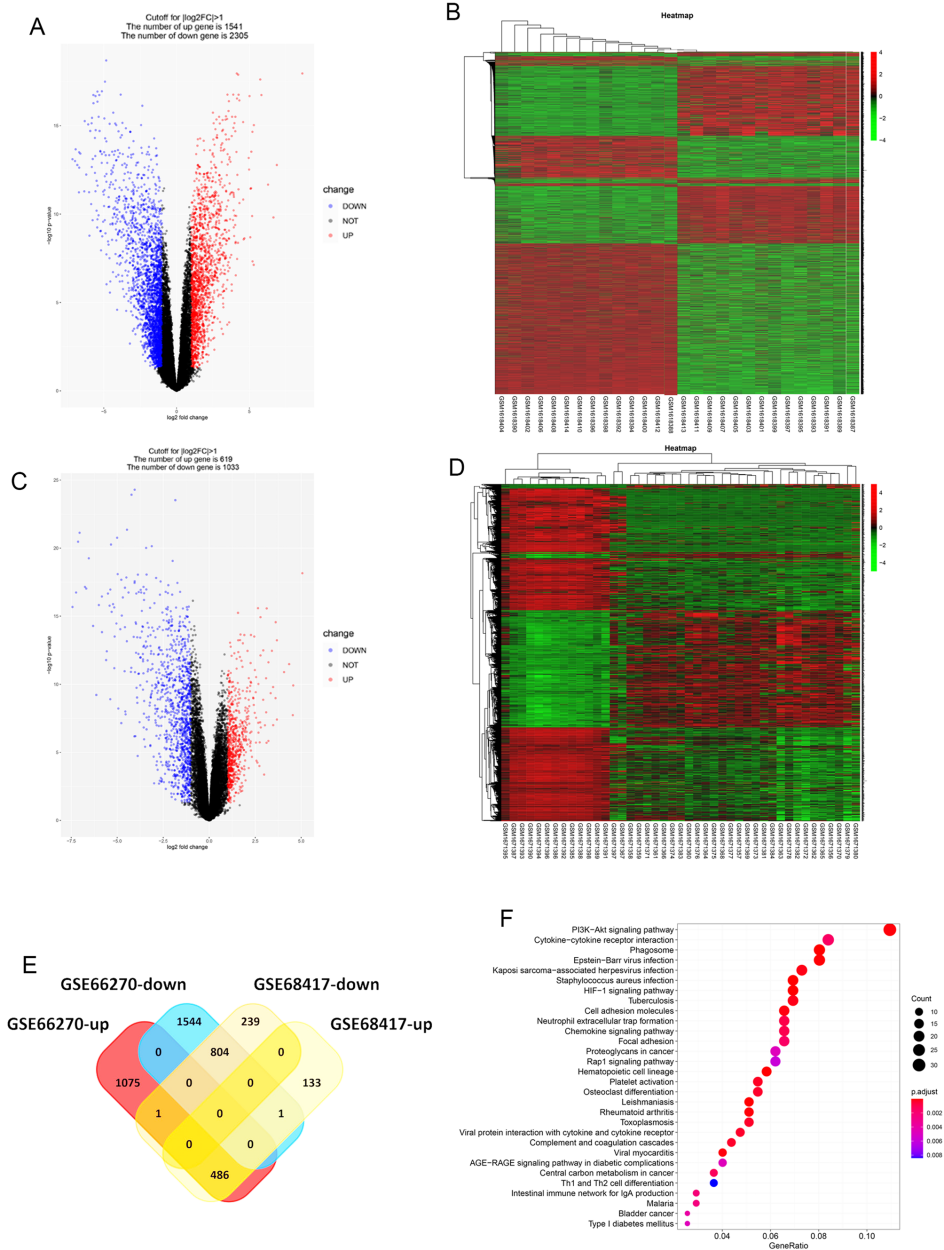
Identification of DEGs in ccRCC. **A–D** Volcano plots and heatmaps of the DEGs in the GSE66270 and GSE68417 datasets. **E** Venn diagrams of the overlapped DEGs. **F** KEGG pathway enrichment analysis

To identify the DEGs in ccRCC, we downloaded the gene expression profile from the GEO database. A total of 3846 DEGs (1541 upregulated and 2305 downregulated) were identified in the GSE66270 dataset (Fig. 1A, B). A total of 1652 DEGs (619 upregulated and 1033 downregulated) were identified in the GSE68417 dataset (Fig. 1C, D). The intersection of the above-mentioned DEGs in a Venn plot yielded 1290 co-DEGs (Fig. 1E). To better understand the co-DEGs involved in ccRCC progression, the KEGG pathway enrichment analysis was performed. The results revealed that these DEGs were significantly associated with the PI3K-Akt signaling pathway and cytokine − cytokine receptor interaction (Fig. 1F). The PI3K-AKT pathway is a crucial signaling pathway in cellular processes such as proliferation and differentiation and cytokine–cytokine receptor interaction involved in tumorigenesis and pathogenesis. Therefore, these co-DEGs participate in the progress of ccRCC and may drive this process.

### ***ATAD2*** was upregulated in ccRCC cells

**Fig. 2:**
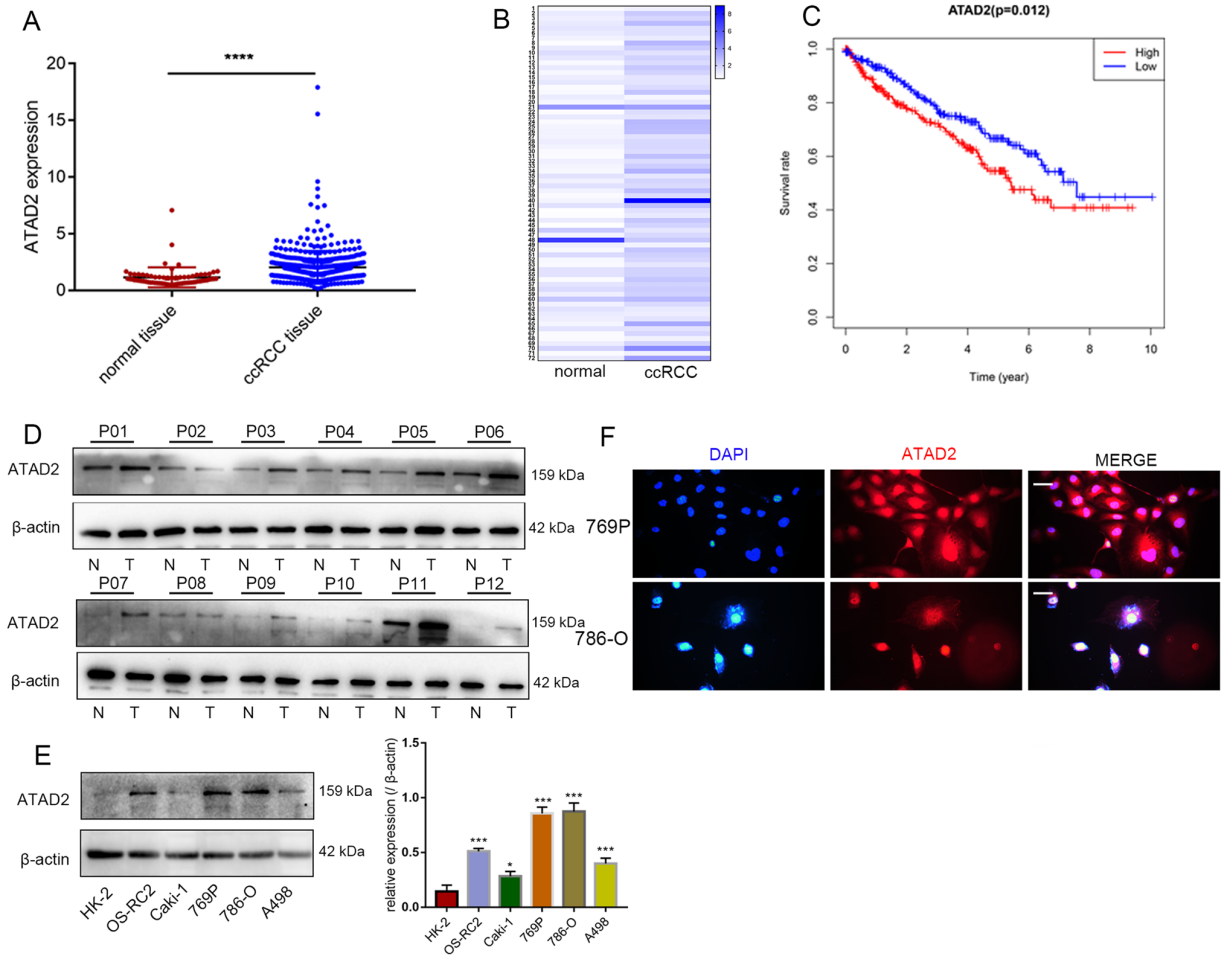
ATAD2 was upregulated in ccRCC. **A** ATAD2 mRNA levels in ccRCC tissues were assessed from the TCGA cohort. **B** ATAD2 mRNA levels in ccRCC samples were compared with those in paired adjacent normal tissues. **C** Kaplan–Meier plots of *ATAD2* expression in ccRCC patients. **D** The expression level of *ATAD2* in 12 paired ccRCC samples (N: normal tissue; T: tumor tissue; P: Patient). **E** ATAD2 protein levels in ccRCC cell lines (786-O, OS-RC-2, 769P, and A498) and HK-2 were assessed by western blotting assays. **F** Localization of ATAD2 was assessed by immunofluorescence. Scale bars represent 20 μm. Data are presented as mean ± SD. ****P < 0.0001

The intersection of the two datasets identified 486 co-upregulated and 804 co-downregulated genes. Among the 486 co-upregulated genes, *ATAD2* is recently reported to be closely associated with tumor progression; however, its role in ccRCC remains elusive. Therefore, we investigated the expression and function of *ATAD2* in ccRCC. Next, we analyzed ATAD2 expression in TCGA and found that the mRNA level of *ATAD2* was significantly increased in ccRCC tissues (Fig. 2A). Similar findings were noted in matched tissues from the same patient (Fig. 2B). In ccRCC patients in TCGA, a high expression of ATAD2 correlated with poor OS (Fig. 2C). 12 pairs of ccRCC tissues and normal specimens were collected. As expected, the ATAD2 protein level also increased in ccRCC tumor tissues (Fig. 2D). Compared to that in HK-2 cells, ATAD2 expression was significantly increased in the ccRCC cell line (Fig. 2E). Immunofluorescence studies revealed that ATAD2 was localized predominantly in the cell nucleus and cytoplasm (Fig. 2F).

### ATAD2 promotes an aggressive phenotype of ccRCC in vitro

**Fig. 3:**
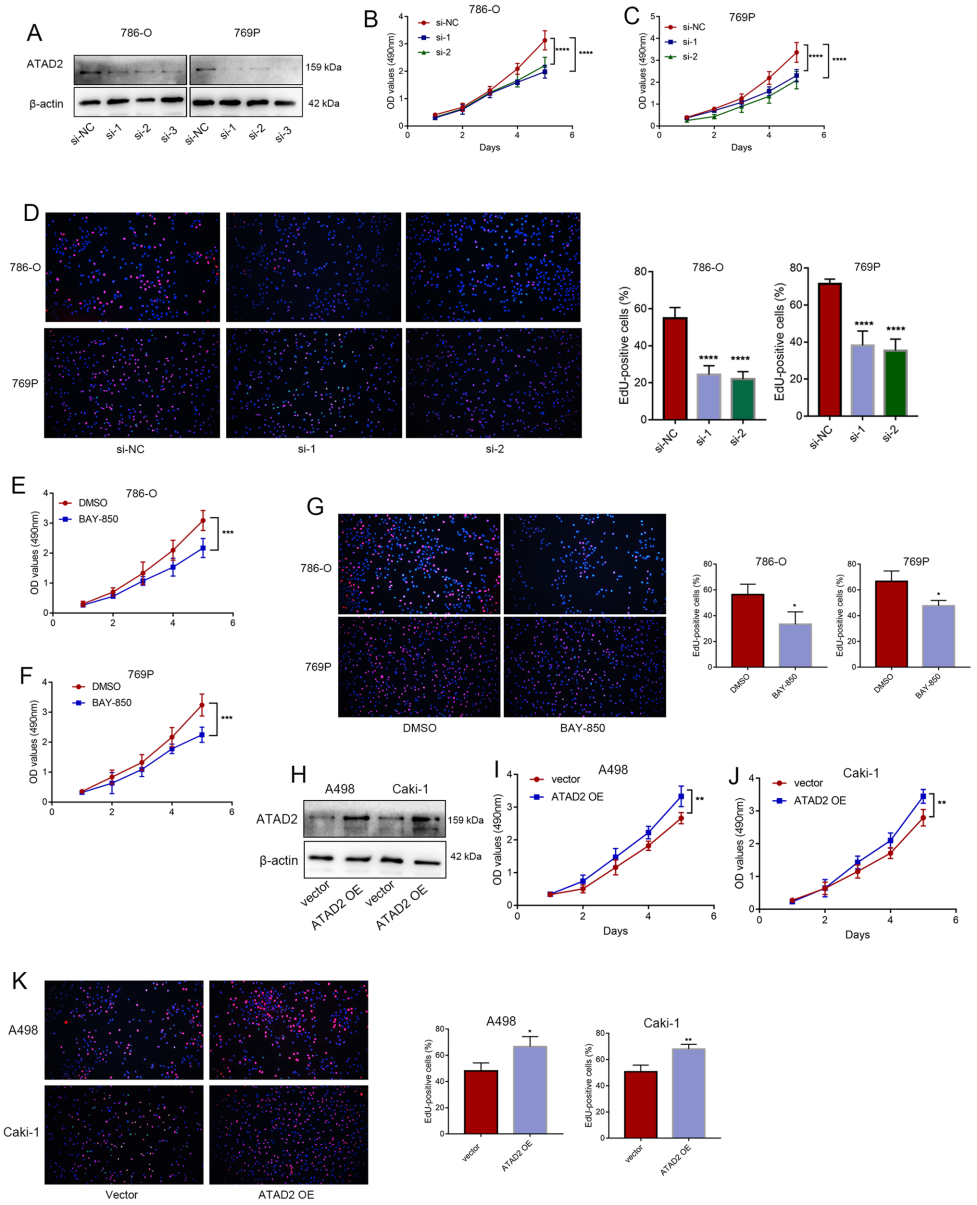
ATAD2 promotes an aggressive phenotype of ccRCCin vitro. **A** Protein expression levels of ATAD2 in 769P and 786-O cells following *ATAD2* knockdown by using siRNAs. **B**–**D** Cell proliferation assays: CCK-8 and EdU assays following *ATAD2* knockdown. **E**–**G** The malignant behaviors of 769P and 786-O cells after treatment with the ATAD2 inhibitor BAY-850.** H–K** The proliferation of A498 and caki-1 cells following *ATAD2* overexpression. Data are presented as mean ± SD. *P < 0.05, **P < 0.01, ***P < 0.001, ****P < 0.0001

We further investigated the role of ATAD2 in the malignant behavior of ccRCC cells. The ATAD2 protein was knocked down in 769P and 786-O cells by using siRNAs (Fig. 3A). CCK-8 and EdU assays showed that inhibiting *ATAD2* expression weakened the proliferation ability of ccRCC cells (Fig. 3B–D). We also found that the ATAD2 inhibitor BAY-850 could suppress the malignant behavior of 769P and 786-O cells (Fig. 3E–G). *ATAD2* was overexpressed in A498 and caki-1 cells through plasmid transfection, and *ATAD2* overexpression significantly enhanced the proliferation of A498 and caki-1 cells (Fig. 3H–K).

### Overexpression of ***ATAD2*** promotes the proliferation of ccRCC cellsin vivo

**Fig. 4:**
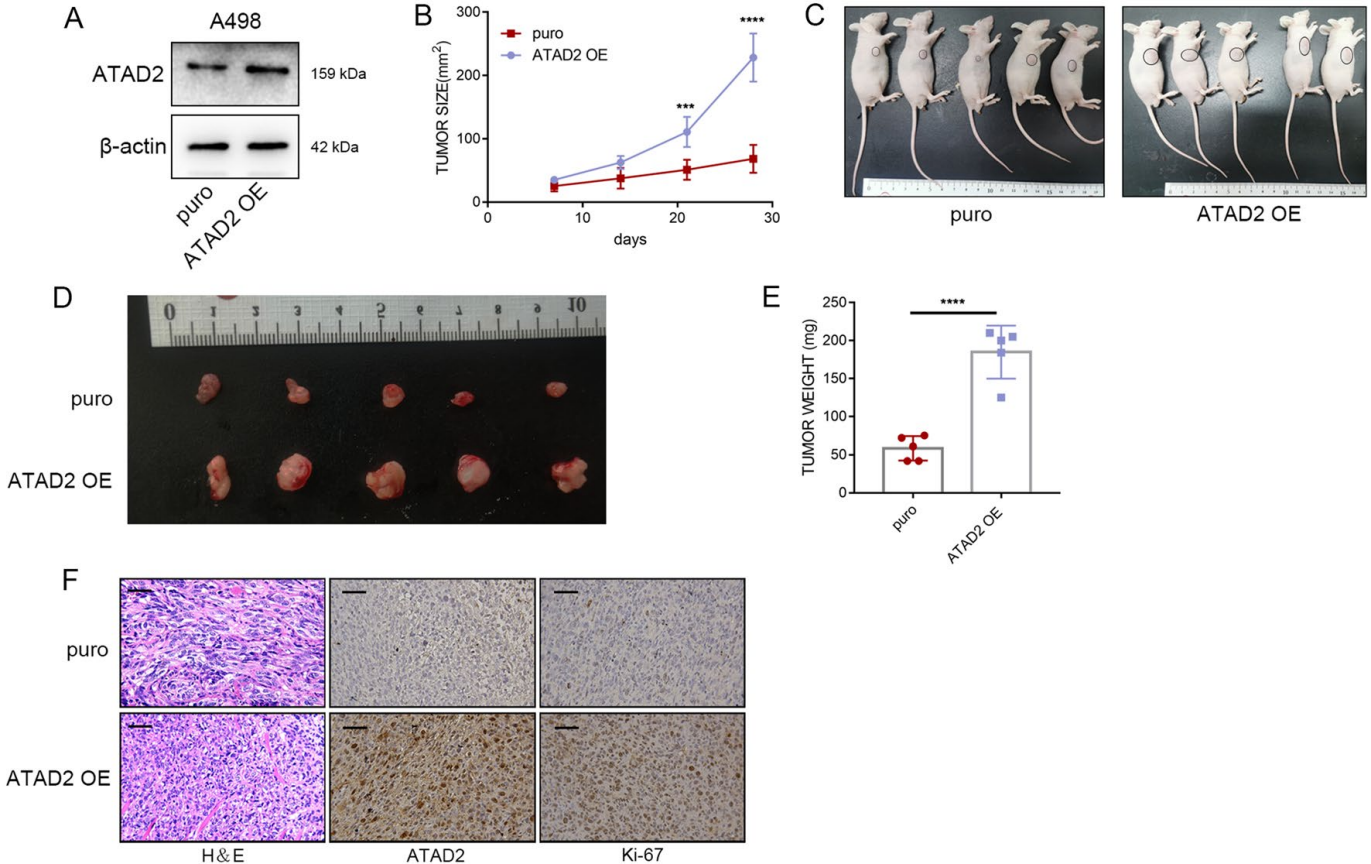
Overexpression of ***ATAD2*** promotes the proliferation of ccRCCin vivo. **A** Protein expression levels of ATAD2 in A498 cells with stable *ATAD2* overexpression. **B** The growth curves of xenografts (n = 5 in each group). **C**, **D** Images of the excised tumors. **E** Tumor weight. **F** Staining of Ki67 and ATAD2. Scale bars represent 50 μm. Data are expressed as mean ± SD. ***P < 0.001, ****P < 0.0001

Cell lines with stable *ATAD2* overexpression were constructed with A498 cells (Fig. 4A). Next, a xenograft model was established. A498 cells were inoculated in the armpit of BALB/c nude mice. *ATAD2* overexpression significantly enhanced tumor growth (Fig. 4B). The nude mice were sacrificed 4 weeks later, and the xenografts were dissected and isolated (Fig. 4C, D). A noteworthy finding was that tumor weights significantly differed between the two groups (Fig. 4E). Moreover, subcutaneous tumor analysis revealed that Ki67 staining abundance was significantly enhanced following *ATAD2* overexpression (Fig. 4F).

### ATAD2 physically interacts with c-Myc

**Fig. 5:**
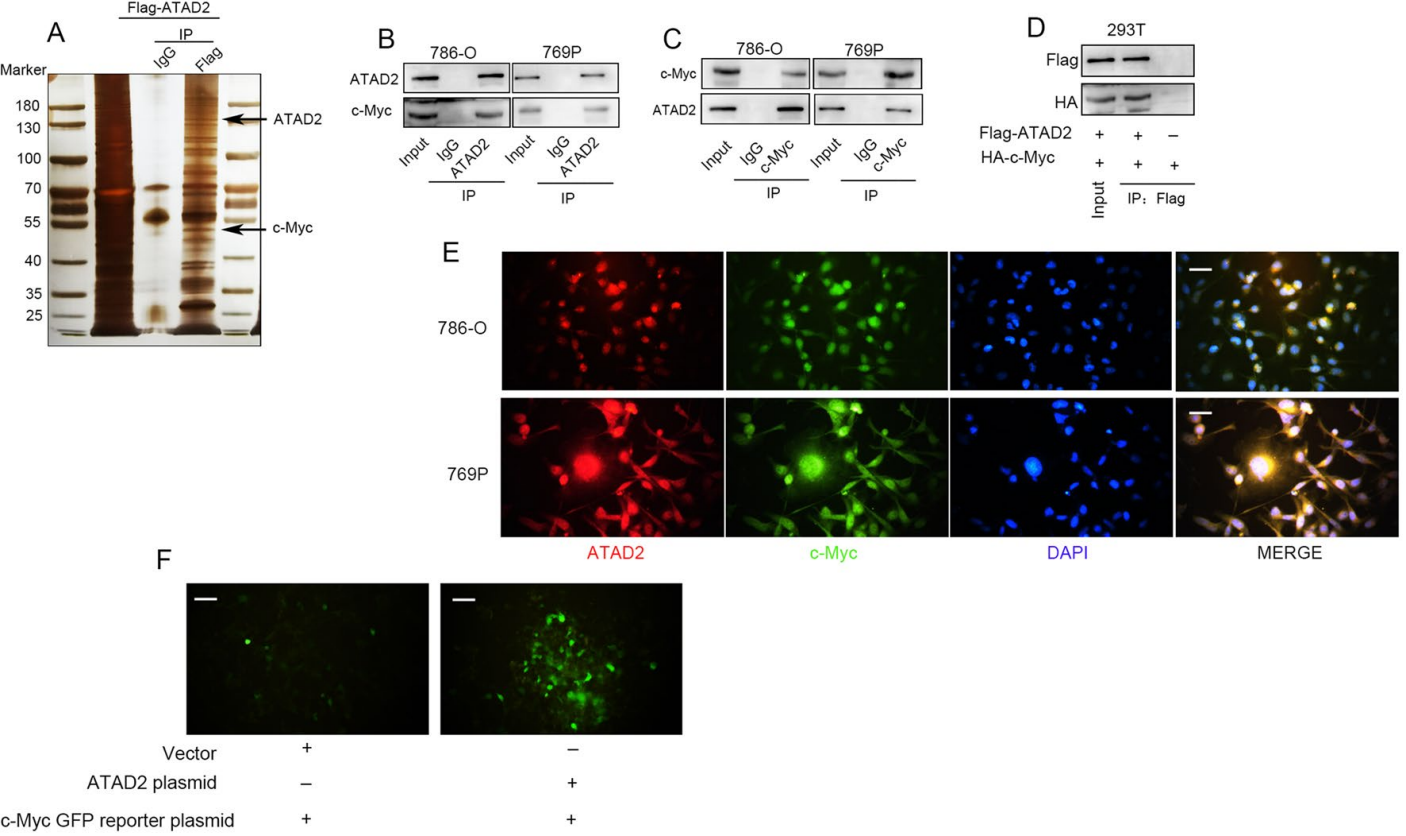
ATAD2 physically interacts with c-Myc. **A** Silver-stained IP sample from Flag-tagged ATAD2 expressed in human HEK293T cells. **B**, **C** Immunoprecipitation (IP) followed by western blotting assay. **D** Co-IP was performed using cell lysates obtained from HEK293T cells co-transfected with Flag-ATAD2 and HA-c-Myc. **E** Double staining of ATAD2 and c-Myc. **F** c-Myc GFP reporter assays. Scale bars represent 20 μm

Next, by using the public database Biogrid [17], which archives information on physical protein-protein interaction, we found that the c-Myc protein interacts with ATAD2. The gene containing the bromodomain domain is considered a mediator of the c-Myc transcription function, and the existence of the bromodomain domain in *ATAD2* implies that it may be a regulator of c-Myc. Silver staining showed a clear band at 159 kDa for ATAD2. A clear single band was also found at 49 kDa, which is consistent with the published molecular weights of c-Myc (Fig. 5A). IP assay of ATAD2 showed c-Myc co-precipitation, and conversely, ATAD2 was present in the co-precipitation of c-Myc (Fig. 5B, C). Next, HEK293T cells were co-transfected with FLAG-tagged human c-Myc and HA-tagged human ATAD2 and subjected to the Co-IP assay. The results demonstrated that these two proteins could physically interact with each other (Fig. 5D). Immunofluorescence analysis revealed a co-localization of ATAD2 with c-Myc in 769P and 786-O cells (Fig. 5E). HEK293T cells were co-transfected with the c-Myc GFP reporter plasmid and an empty vector plasmid or a plasmid expressing ATAD2. Co-transfection of the c-Myc GFP reporter plasmid and the ATAD2 overexpression vector significantly increased GFP expression; this finding suggested that ATAD2 enhanced the downstream transcriptional activity of c-Myc (Fig. 5F).

### ATAD2 promotes glycolysis in ccRCC cells

**Fig. 6:**
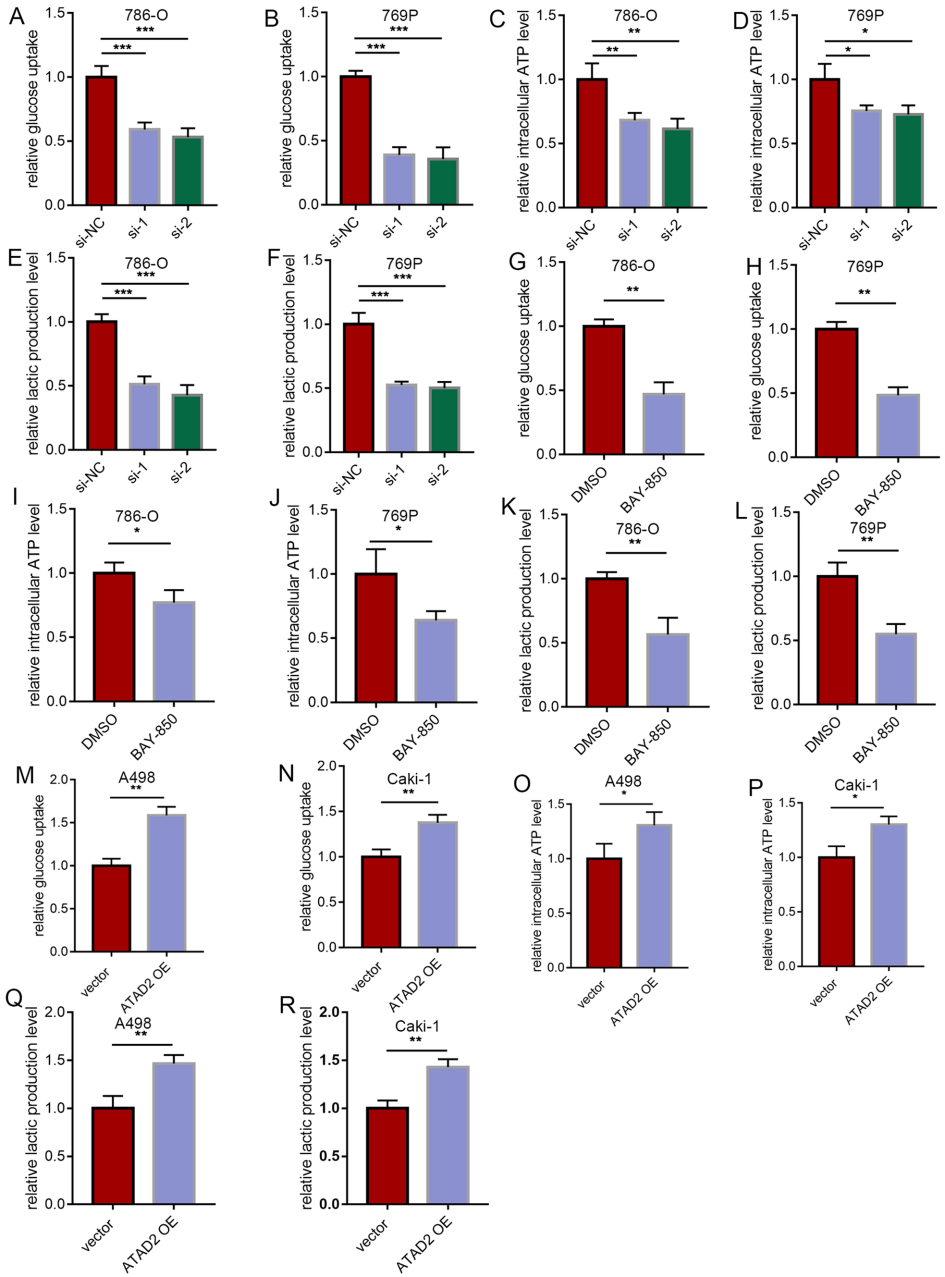
ATAD2 promotes glycolysis in ccRCC cells. **A–F** Glucose uptake, ATP content, and lactate level in ccRCC cells after *ATAD2* knockdown. **G–L** Glucose uptake, ATP content, and lactate level in ccRCC cells after treatment with BAY-850. **M–R** Glucose uptake, ATP content, and lactate level in ccRCC cells after *ATAD2* overexpression. Data are expressed as mean ± SD. *P < 0.05, **P < 0.01, ***P < 0.001

Because c-Myc enhances glycolysis without hypoxia and alters tumor cell metabolism, we speculated that ATAD2 cooperates with c-Myc to promote glycolysis in ccRCC cells. To clarify whether ATAD2 promotes glycolysis in ccRCC cells, we measured glucose intake, ATP content, and lactate level. *ATAD2* knockdown or treatment with BAY-850 decreased glucose uptake, ATP content, and lactate level in ccRCC cells (Fig. 6A–L), while *ATAD2* overexpression increased glucose uptake, ATP content, and lactate level in ccRCC cells (Fig. 6M–R).

### ATAD2 contributed to the glycolysis pathway activation

**Fig. 7:**
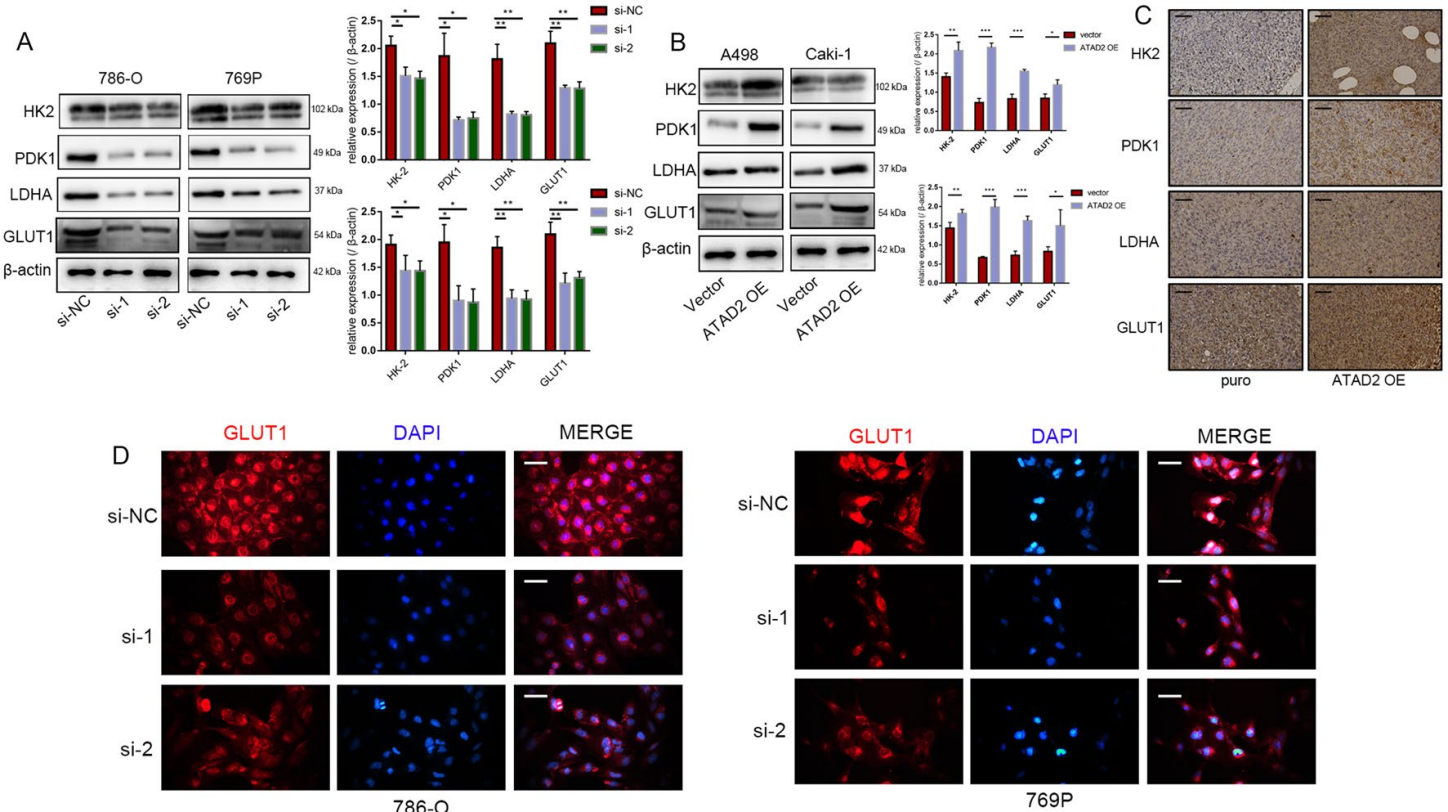
ATAD2 contributed to the activation of the glycolysis pathway. **A**, **B** The expression level of key genes in glycolysis (HK2, PDK1, LDH, and GLUT1) after knockdown or overexpression of *ATAD2*. **C** The expression of glycolysis-related markers in subcutaneous xenograft tumor tissues of nude mice. Scale bars represent 50 μm. **D** Fluorescence intensity of GLUT1 after *ATAD2* knockdown. Scale bars represent 20 μm

GLUT1 is a well-known key glucose carrier; its expression is upregulated in different cancers and is associated with poor overall survival rates. HK2, PDK1, and LDHA are also reported to be key participants in the tumor glycolysis process. We found that *ATAD2* knockdown decreased the levels of GLUT1, HK2, PDK1, and LDHA, while *ATAD2* overexpression enhanced the expression of these key genes in glycolysis (Fig. 7A, B). Consistently, we also found that the expression of glycolysis-related markers in subcutaneous xenograft tumor tissues of nude mice exhibited the same trend as that noted in in vitro experiments (Fig. 7C). The immunoassay showed that the fluorescence intensity of GLUT1 decreased in cells with *ATAD2* knockdown. A noteworthy finding was that GLUT1 was located in the cell membrane in the control group; however, the cell membrane distribution of GLUT1 in ccRCC cells decreased after *ATAD2* knockdown (Fig. 7D).

### Inhibition of c-Myc reversed the ATAD2-mediated glycolysis in ccRCC cells

**Fig. 8:**
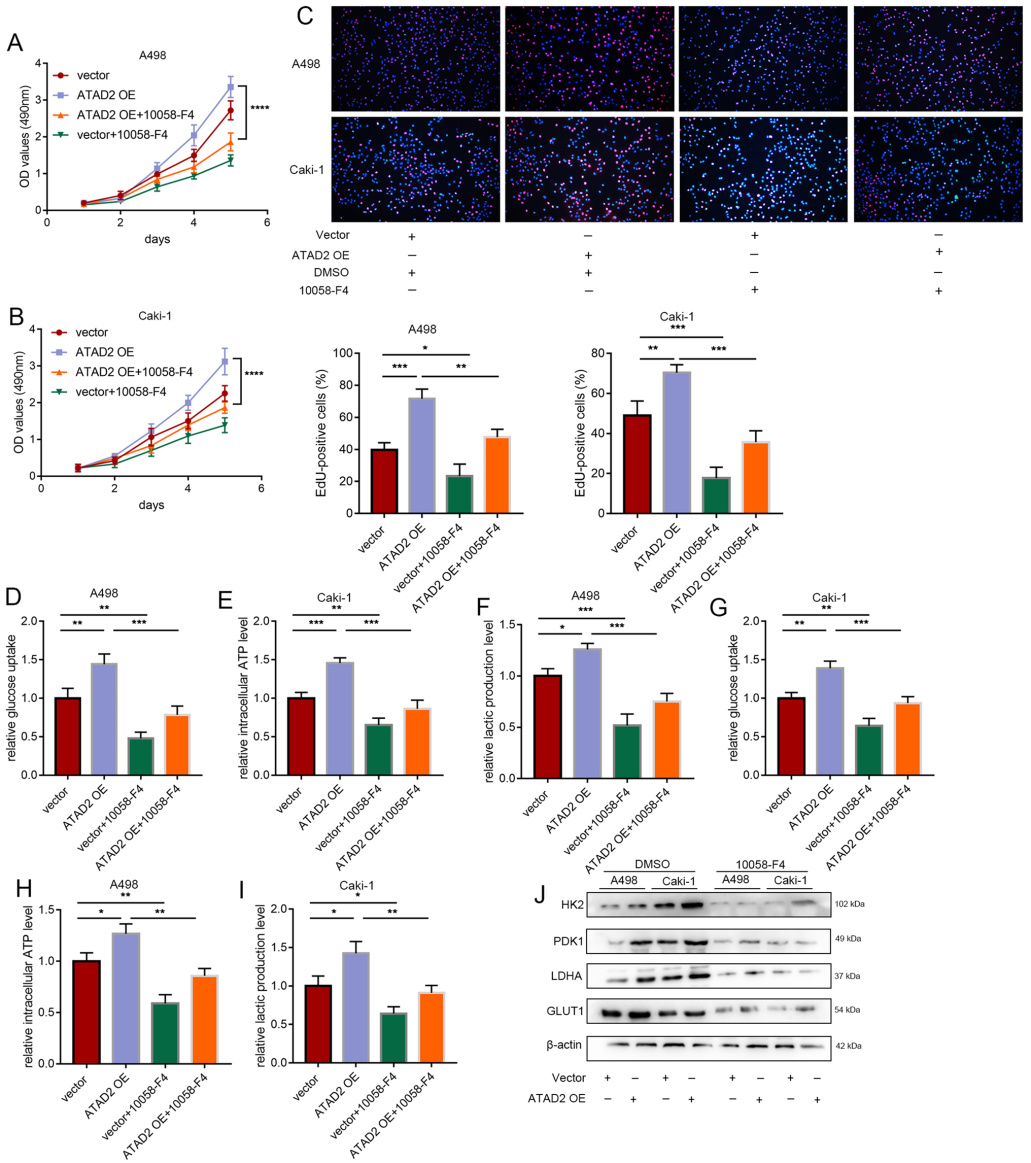
Inhibition of c-Myc reversed ATAD2-mediated glycolysis in ccRCC cells. Rescue experimental paradigm with the c-Myc inhibitor 10,058–F4. ATAD2-overexpressing 786-O and 769P cells after treatment with 10,058–F4 (100  mmol/L) for 24 h: **A–C** Cell proliferation assays: CCK-8 and EdU assays. **D–I** Glucose uptake, ATP content, and lactate level in ccRCC cells. **J** Expression of HK2, PDK1, LDHA, and GLUT1 in ATAD2-overexpressing 786-O and 769P cells after treatment with 10,058–F4

Next, we performed rescue experiments by using 10,058–F4, a specific c-Myc inhibitor. c-Myc inhibition partially reversed the effects of *ATAD2* overexpression in promoting the malignant behavior and glycolysis of ccRCC cells (Fig. 8A–I). The upregulation of HK2, PDK1, GLUT1, and LDHA induced by *ATAD2* overexpression was weakened by treatment with 10,058–F4 (Fig. 8J). Overall, our results implied that the effect of ATAD2 was mediated by the increase in the transcriptional activity of c-Myc.

## Discussion

Radical nephrectomy remains the mainstay of curative treatment for patients with ccRCC. However, approximately 20–30% of patients show distant metastasis at the initial diagnosis stage [18]. Although molecular targeted drugs and immunotherapy have improved the survival of patients with metastasis, the overall prognosis remains poor [19]. Most cancer cells exhibit alterations in their energy metabolism pathways, even in the presence of oxygen, and they produce lactic acid through glycolysis. Glycolysis is a critical pathway for tumor cells to obtain energy, and the key enzymes involved in glycolysis are highly expressed in tumor cells to produce more energy. The fermentation of lactic acid, a glycolysis product, also provides energy to tumor cells [20]. Interestingly, the Warburg effect is more apparent in ccRCC than in other tumors [21]. Glycolysis is active in ccRCC cells, and it represents an important feature of ccRCC as metabolites of the glycolysis pathway increase with a decrease in oxidative phosphorylation [22]. ccRCC is even considered a metabolic disease because of its excessive glycogen and lipid deposition in the cytoplasm [23–25]. Therefore, in our search for potential therapeutic targets and prognostic biomarkers, the investigation of the molecular mechanisms underlying the occurrence and progression of renal cell carcinoma is essential.

The abnormal expression of *ATAD2* is closely associated with the occurrence and development of various tumors. In lung adenocarcinoma, *ATAD2* expression is associated with patient gender, smoking status, tumor stage, and lymph node metastasis and correlates with a high risk of recurrence [26]. There is an increasing consensus that ATAD2 is closely related to the activation of some proto-oncogenes by acting as a co-activator of some transcription factors to regulate the expression of genes with carcinogenic function[8, 27, 28]. However, the regulation of ATAD2 and its related mechanisms remain elusive. In the present study, we found that *ATAD2* was upregulated in ccRCC and promoted an aggressive phenotype of ccRCC. We also observed that ATAD2 promoted glycolysis in ccRCC cells and influenced glucose intake, ATP content, and lactate level. *ATAD2* knockdown decreased the expression levels of GLUT1, HK2, PDK1, and LDHA, while *ATAD2* overexpression increased the expression of the key genes in glycolysis. This process depends on the direct binding of ATAD2 with c-Myc, which promotes c-Myc activation and transcription of its downstream genes.

c-Myc upregulates various glycolytic enzymes such as GLUT-1, HK2, PDK1, and LDHA and promotes glycolysis under normal oxygen conditions [29–31]. During glycolysis, GLUT1 is a rate-limiting enzyme that promotes glucose transport across the cell membrane [32]. Current evidence suggests that GLUT1 overexpression, particularly on the cell membrane, is closely associated with the malignant progression of tumors [33]. HK2 is a hexokinase that participates in glycolysis and is essential for the Warburg effect. HK2 level increases in several types of human tumors, and this enzyme participates in the cell cycle process [34–36]. PDK1 phosphorylates the pyruvate dehydrogenase (PDH) E1α subunit to inactivate the PDH enzyme complex, and the PDH enzyme complex can then convert pyruvate into acetyl-CoA. Therefore, PDK1 overexpression provides a source of pyruvate for glycolysis [37, 38]. LDHA is the key glycolytic enzyme that catalyzes pyruvate to lactic acid during the last step of anaerobic glycolysis [39]. LDHA is abnormally expressed in many cancers, including pancreatic cancer, hepatocellular carcinoma, and breast cancer [40–42]. LDHA plays a potential role in the pathogenesis and maintenance of tumorigenesis and malignant transformation [43]. In the present study, the membrane distribution of GLUT1 in ccRCC cells was decreased after *ATAD2* knockdown. Moreover, *ATAD2* knockdown also decreased the expression level of GLUT1, HK2, PDK1, and LDHA, while *ATAD2* overexpression enhanced the expression of these key genes in glycolysis. An important finding is that ATAD2 is a cofactor of c-Myc and enhances its downstream transcriptional activity, thereby promoting glycolysis in ccRCC.

Overall, our results reveal that *ATAD2* expression is dysregulated in ccRCC cells. We found that ATAD2 regulated the transcriptional activity of c-Myc and promoted the expression of the glycolytic genes and the Warburg effect. Because the Warburg effect plays a critical role in promoting tumor growth, our data further support that ATAD2 has a huge potential to function as a therapeutic target for treating ccRCC.

## Supplementary Information

**Additional file 1**. The list of antibodies used in the experiment

## Data Availability

Data available within the article or its supplementary materials.
