# Supplementary material for: ATAD2 promotes glycolysis and tumor progression in clear cell renal cell carcinoma by regulating the transcriptional activity of c-Myc
Source: Discov Oncol. 2023 May 26;14:79. doi: 10.1007/s12672-023-00696-1 (PMC10219916; doi:10.1007/s12672-023-00696-1)
Supplement: Supplementary file 1 — Additional file 1. The list of antibodies used in the experiment [file 12672_2023_696_MOESM1_ESM.docx]

**Supplemental Table S1. The list of antibodies used in the experiment**

| ATAD2 | 1:1000, Proteintech, 23894-1-AP |
| --- | --- |
| c-MYC | 1:1000, Proteintech, 67447-1-Ig |
| HK2 | 1:1000, Proteintech, 22029-1-AP |
| PDK1 | 1:1000, Proteintech, 18262-1-AP |
| LDHA | 1:1000, Proteintech, 21799-1-AP |
| GLUT1 | 1:1000, Proteintech, 21829-1-AP |
| β- Actin | 1:1000, Proteintech, 81115-1-RR |
